# Barriers and opportunities for improving childhood immunization coverage in slums: A qualitative study

**DOI:** 10.1016/j.pmedr.2019.100858

**Published:** 2019-03-28

**Authors:** Sanjeev Singh, Damodar Sahu, Ashish Agrawal, Meeta Dhaval Vashi

**Affiliations:** a University School of Medicine & Paramedical Health Sciences, Guru Gobind Singh Indraprastha University, Delhi, India; b GlaxoSmithKline Pharmaceuticals Ltd, India; c National Institute of Medical Statistics (NIMS), Indian Council of Medical Research (ICMR), Delhi, India; d National Polio Surveillance Project, Mumbai, Maharashtra, India

**Keywords:** Immunization, Vaccine, Vaccination, Slums, Qualitative, India

## Abstract

There is substantial variability in immunization coverage trends across the globe which can be attributed to a number of factors such as demographic profile, socioeconomic characteristics and political environment. Vaccine preventable diseases contribute to severe disease burden when coverage is low, particularly, in slums. Present qualitative study explored barriers, opportunities, and key facilitators of childhood immunization. This was a community based cross-sectional study conducted in the slum areas of Mumbai, India. Data from the observations of immunization sessions and interviews of end users, healthcare service providers, and influencers were collected and analyzed. Lack of time, poor awareness, fear of adverse event, loss of daily income, and migrant population were some of the major reasons to not get immunized. Also, lack of good behavior of staff was another crucial factor perceived by caretakers as barrier in the immunization. Stakeholders agreed that immunization is a shared responsibility involving community, service providers, and policy makers. There was general consensus that immunization practices have improved over the last few years. However, its positive impact is yet to be fully seen in populations that belong to lower socioeconomic strata, thus warranting additional efforts to improve the immunization coverage in slums. Effective communication, process improvement at various levels, active involvement of communities in the immunization activities, building trust and accountability, and constructive feedback are some of the essential elements to strengthen the immunization program. Strategies to improve immunization services in such settings should be based on interactions with stakeholders and understanding their perspectives.

## 1 Introduction

Immunization, one of the most cost-effective preventive healthcare intervention has moved to center stage and is a driving force in reducing child mortality, especially by controlling vaccine preventable diseases (VPDs) (Duclos et al., 2009). Ensuring high immunization coverage and its acceptance among the beneficiaries is crucial for a healthy society. Immunization is a multi-sectorial activity, and a substantial variability in coverage exists across the globe influenced by varying demographic, socioeconomic and political structures (Singh, 2018). Also, factors like education, occupation, household income, gender, living condition, habitation, awareness, religion, etc. appear to play a significant role even in the presence of no cost immunization program and other healthcare services (Kulkarni and Chavan, 2013). It is a well-known fact that when immunization coverage is low, VPDs contributes to worse health outcomes, particularly in slums (Crocker-Buque et al., 2017). Situation is perilous for India as nearly 33% (100 million) of the urban population lives in unorganized slums. The slum dwellers are characterized as one of the most vulnerable populations to outbreaks of VPDs due to overcrowding, scarcities in the healthcare system, poor hygiene and improper sanitation (Agrawal et al., 2014; Singh, 2018).

Existing immunization practices and delivery systems do not effectively meet the needs, especially for those living in slums, resulting in lower coverage. Despite many efforts, inhabitants living in these settings present a challenge for the attainment of the national goals. A mere inclusion of vaccines in the national immunization program is not sufficient. A special emphasis on its effective implementation in low socioeconomic and unorganized areas is essential (Singh, 2018).

Research on immunization is fragmented. Most of the studies are based on quantitative approaches, which have their own limitations to provide detailed views of the various stakeholders and complex contexts within the community that impact the coverage or other desired outcomes related to the immunization program (Adam et al., 2015; Babirye et al., 2011; Sinuff et al., 2007). For example, interventions such as reminders or recall, financial incentives, reducing the physical distance to health services and regular monitoring can be driving forces to accelerate immunization services (Singh, 2018).

A multi-level qualitative approach offers an opportunity to have real time interactions with stakeholders such as beneficiaries, healthcare service providers, policy makers, and influencers who form a principal element of any immunization program. This will enable to understand their unbiased perspectives, which can have an ability to influence the coverage or other desired outcomes. Evidences generated through this approach are key to understand the underlying potential drivers, parents' reservations to not immunize their children, immunization needs of the slums, and to analyze the levels of influence for health-related behaviors (Babalola, 2011; Bingham et al., 2009). Our study aims to explore major barriers, potential opportunities, and key facilitators of childhood immunization in slums by using qualitative approach.

## 2 Methods

### 2.1 Study site

**Fig. 1:**
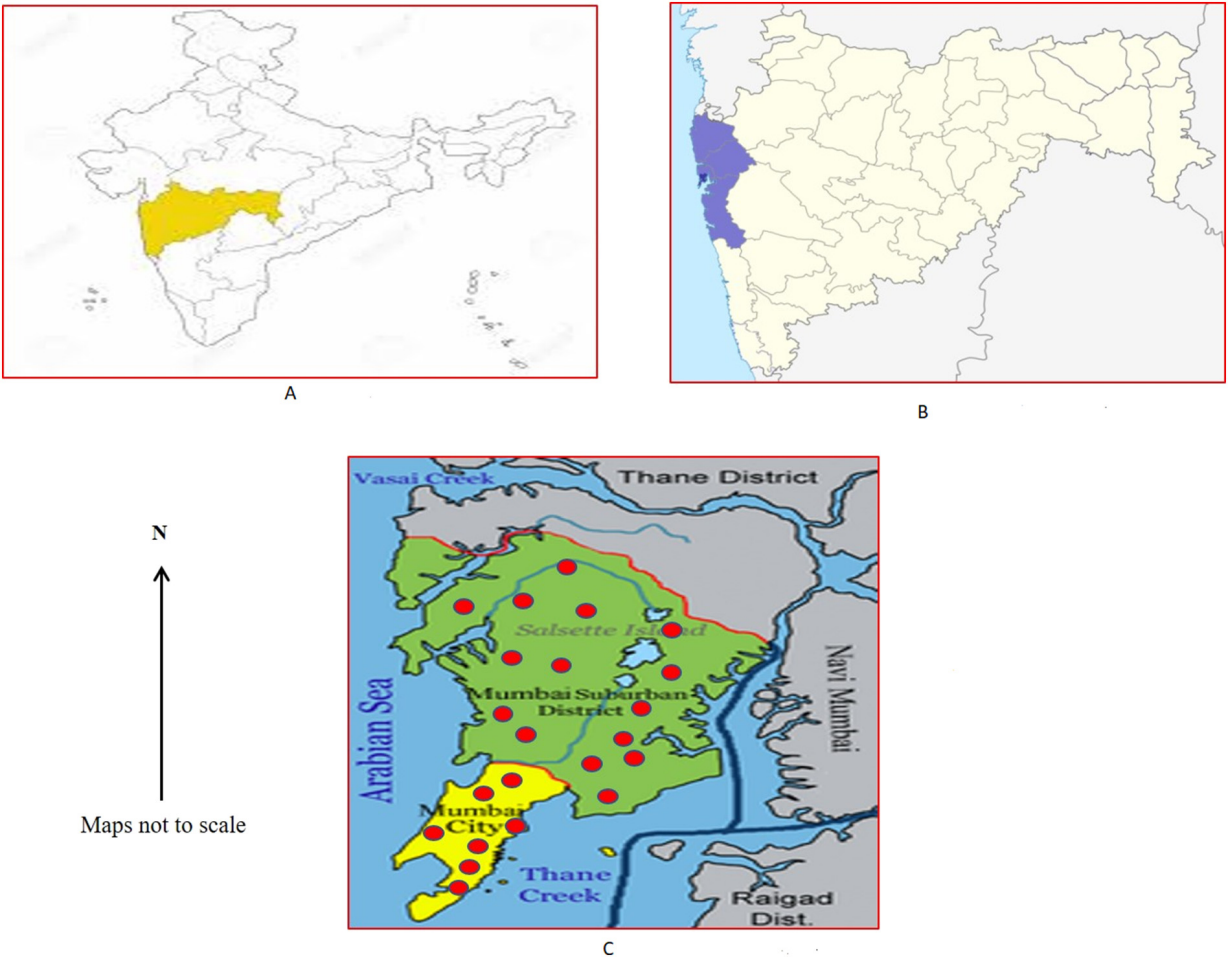
Study location. A: Location of Maharashtra State in India. B: Location of Mumbai in India. C: Study districts and sites in Mumbai metropolitan region.

For this study, slum was defined according to the census of India as *“a residential area where dwellings are unfit for human habitation by reason of dilapidation, overcrowding, faulty arrangements and design of such buildings, narrowness or faulty arrangement of streets, lack of ventilation, light, or sanitation facilities or any combination of these factors which are detrimental to the health and safety of the inhabitants”* (Census of India, 2011). Mumbai is among the world's most populous cities with approximately 20.5 million inhabitants with 62% living in slums. The growing density of the slum population is 334,728 per square kilometer (WPR-Mumbai Population) which makes it vulnerable to disease outbreaks. The study was conducted in the health posts areas of Mumbai City (n = 52) and Mumbai suburban districts (n = 153) in Maharashtra state, India (Fig. 1). Health Posts was defined as “*set up in the community to deliver preventive and promotive health care services and is expected to cover a population group that has 40 percent of its constituents living in slum/slum like localities*” (Dilip and Duggal, 2004). Each of these health post areas consists of 4–5 health workers (Assistant medical officer, auxiliary nurse midwife, public health nurse and coordinator). A multistage cluster sampling method was used for identifying and better representation of all the health posts, considered as study clusters. Fifty-five slum health posts (Mumbai city: 13; Mumbai suburban district: 42) out of total 205 health posts (distributed in both districts) were randomly selected as clusters using the ‘probability proportional to size (PPS) technique’. This method was adopted to get 550 samples (10 samples from each cluster) for the quantitative part of the study. We use the same clusters (n = 55) for the qualitative data collection.

### 2.2 Study design

This was a community based cross-sectional study that used quantitative and qualitative approach. Here, we present the data from the qualitative analysis, non-participatory observations (NPOs), and in-depth interviews (IDI). This design will allow the researchers to build a holistic, detailed description, and analysis of the factors associated with routine childhood immunization within its real-world context (Baxter and Jack, 2008; Bingham et al., 2009). The study was approved by the Institutional Ethics Committee of NIMS, ICMR, New Delhi, India.

### 2.3 Study samples

To meet the objective of the study, purposive sampling was done in the 55 clusters which had earlier been selected randomly using PPS technique. Different stakeholders who were actively involved at various levels of immunization related activities were interviewed:

• NPOs of immunization sessions = 10
• IDIs of influencers in the family (fathers, mothers, and grandparents, n = 50), healthcare service providers (medical officer, district immunization officer, district health officer, n = 12) policy makers (state health officer, n = 1), and policy influencers (international agencies working on immunization, n = 2) = 65.

### 2.4 Data collection and analysis

We used a semi-structured questionnaire for IDIs. and a predefined guideline to document observations during the NPOs. All IDIs were transcribed verbatim and translated from local languages into English. The transcribed data were reviewed and after several reviews, key themes and sub themes were identified based on the objective of the study. In this study, we used thematic analysis, incorporating an explanation of the elements explored in-depth. Ongoing data analysis during the study process allowed the authors to condense an extensive amount of information and its verification into a more manageable format (Lincoln and Guba, 1985; Strauss and Corbin, 1998). To organize the data, we also used comparison table to compare views of groups of caretakers on one theme and demographic table of participant numbers across the study clusters (Yin, 2003). Our analysis involved organizing data, breaking them into more manageable categories, developing codes, and searching for possible patterns for a comparative perspective using ATLAS.ti software, version 7 (Friese, 2013).

## 3 Results

### 3.1 Non-participatory observation of the immunization session

The observations were made after obtaining permission from the facility. On an average 2–3 workers were present during each session. The overall observations are presented in Table 1. It was observed that vaccines were stored appropriately, and child received vaccines appropriate for its age. However, emergency medicines for managing potential adverse events (AEs) were available only at few centers (30%). Specific instructions for the vaccine the child received was not provided to mothers or caregivers. They were apprised with limited information with respect to the next immunization and the possibility of adverse events such as fever, pain, crying, swelling following immunization. Immunization related information displayed on the walls at health facilities was outdated. For example, posters on national pulse polio program, hepatitis B, and measles immunization. Further, most of the times the mothers or the caregivers had to wait for a long time before their child received vaccine. This was attributable to several factors such as staff arriving late, vial not opened before a certain number of children reached, poor interaction between the mothers and healthcare workers, etc. Also, the attitude of healthcare workers towards the beneficiaries was quite variable. Some were warm and friendly but majority of them were authoritative demonstrating lack of empathy. This could be attributed to the fact that the work load was quite substantial, and health posts were understaffed.

**Table 1:** Non-participatory observations at health facility.

Observations	Overall observations	
	Favorable	Unfavorable and needs attention
Pre-immunization	• Session sites clean and arranged • Proper sitting arrangement • Vaccines appropriately stored • Uninterrupted electric supply • Proper cold chain	• Session started late • Conditioning of ice packs not performed • Emergency medicines not available • Adverse Effect Following Immunization (AEFI) safety kit
During-immunization	• Child received appropriate vaccines • Appropriate injection site and route • Appropriate diluent • Sufficient time to beneficiary/care taker	• Vaccinators touched or recap the needle • Removing of air bubbling from AD syringes • Cleaning of injection sites with spirit • Post injection, applying pressure over injection site • Real time reporting and recording not done
Post-immunization	• Mothers told to wait (15–30 min) after the immunization of their kids • Immunization recorded on health card and handed over to mothers	• Mothers/care takers not told about next dose • Safety i.e. possibility of fever, pain, etc. not communicated to the beneficiaries • Prophylaxis for fever or pain not given • Improper discarding of used vials and syringes

There is a potential scope for improvement and creating awareness with respect to the immunization, its importance, the diseases they protect, and the overall benefits to the child by replacing the old and outdated immunization related display materials. Self-playing videos in local languages on various health topics including immunization could generate more interests among mothers and caregivers during their waiting time for the immunization. Health talks by service providers on VPDs, immunization, nutrition, etc. during pre and post immunization sessions could be one of the options to create awareness among mothers or caregivers. Healthcare services with emotion and personal touch could strengthen the relationship between the healthcare service provider and the beneficiary.

Though the staff was technically qualified following observations were made: inadequate/improper removal of air bubbles from the Auto-Disable (AD) syringes, use of spirit for cleaning the injection site, pressing the site post immunization, touching and bending needles, etc. Documentation of immunization record, loading and handling techniques of vaccines were also found to be inadequate. On the job trainings, orientations, and supportive supervision which were found to be missing could be key in bringing about improvement in this area. In addition, sensitizing healthcare service providers about their contribution in improving immunization coverage could be motivating factor to perform.

### 3.2 In-depth interview of the influencers

Total of 50 influencers in the family were interviewed and their observations are summarized (Table 2). Majority (93%) of the influencers did not have any idea of the VPDs, however they knew about polio drops and to some extent of an injection that left a scar on the left upper arm. They did not know that it was Bacillus Calmette Guerin (BCG) vaccine. Also, most of the influencers did not know about the vaccines covered in the national immunization program (NIP) and its schedule. Participants informed that they received immunization related information from relatives, neighbors, television or newspapers. They were aware of the vaccination centers and also informed or advised others (e.g. neighbors, friends, etc.) to get their children vaccinated.

**Table 2:** In-depth interview of the influencers (mother, father or grandparents).

Area	Key observations	
Knowledge about VPDs	• Majority did not had any idea except for polio.	
Strategies to prevent VPDs	• Cleanliness • Healthy food, exercises and clean water • Proper drainage system • Immunization	
List few important vaccines	• Majority polio, some talked about BCG and measles	
Perceptions about vaccines as a healthcare service	• Important, prevent diseases, and makes kids healthy • Good for kid's health but no quick effect • Recommended immunization services, especially, to neighbors at least once	
Major challenges with respect to vaccination services in the slum	• Don't take it seriously vs water, food, etc. • Had to wait long	• Good behavior of staff missing sometimes • Need education about benefits
Reasons for not vaccinating	• No time • Experienced adverse event (e.g. fever, pain) • Not informed	• Not important • Work or event in family • Fear
Opportunities available to improve the vaccination?	• Weekly announcement • Recall message by text or by call • One-day prior door to door visit • Incentives e.g. for travel • Immunization session at evening or weekends • Clubbing immunization services with other health services • Award for those who completes all the vaccines on time • Regular community and healthcare service provider meeting	
Expectations from and service providers/healthcare system?	• Be polite • On time services • Inform and educate	• Visit at least once per week • Offer other services • Monitor session and take feedback

One of the participants narrated, “*There is scarcity of everything in our slum e.g. safe drinking water, good quality of air, etc. Therefore, we have to be extra cautious about the safety of our kids. Immunization plays an important role in these conditions. Thus, we must vaccinate our kids and advocate it to others as well*.”

Another participant stated, “*If government is thinking about the improvement of our health then why should we not think about our kids*.”

Lack of time, unawareness, long waiting hours, fear of adverse event, concern related to loss of daily income were some of the major challenges or reasons for not getting their children vaccinated. Also, poor/rude behavior of staff was an important factor that led to either delayed or missed immunization.

One of the participant stated, “*We want to vaccinate our kids, but we fear as some people said that it has risk and may cause disability*.”

Another participant said, *“The last time fever and pain persisted for one week and we were lost as no one was there to respond. I do not want to vaccinate my kid again.”*

One participant said, “W*e want to vaccinate our children, but healthcare service providers have to guide, educate, respect, and at least be sensitive to our issues and challenges like family problem, loss of wages, on time services, other priorities, etc.”*

Another participant stated, *“I hesitate to get vaccinated or to advocate it as I am a non-Marathi, nonlocal. I am from a different state where culture and languages are completely different. I do not know what the local healthcare service providers will think and how they may behave.”*

Most of the influencers expressed that current situation could be improved by:

• Improving condition of healthcare facilities
• Strengthening trust between healthcare service providers and beneficiaries
• Bringing accountability and emotions in the healthcare services
• Involving communities during immunization services
• Creating ongoing awareness among parents and caregivers- conducting various campaigns similar to that for pulse polio program
• Incentives for providing and getting immunization services
• Text message or recall for the upcoming/pending doses
• Timeliness, completeness, and quality in services
• Community feedback on immunization services

### 3.3 In-depth interview of healthcare service providers, policy makers, and policy influencer

Total 15 key participants at various levels were interviewed using a pre-structured questionnaire. Few key questions and their responses are given below:

#### 3.3.1 Do service providers and beneficiaries understand VPDs, vaccines, and importance of vaccination specific to slum settings?

Auxiliary nurse midwives, supervisors and other service providers are trained, accountable and responsible to vaccinate. They understand the importance of the immunization. Also, introduction of newer vaccines into the NIP such as measles-rubella (MR), rotavirus, pneumococcal conjugate vaccine (PCV), etc. and campaigns by the government at various levels have had a huge impact in creating awareness. Non-government organizations and international agencies like World Health Organization (WHO) and United Nation Children's Fund (UNICEF) are also providing support in improving the immunization coverage. The number of beneficiaries getting vaccinated has significantly improved because of “Mission Indradhanush” launched in December 2014 by the Government of India (GoI) to improve the immunization coverage in the country. People now enquire about vaccines.

#### 3.3.2 How slums are different from other settings? In terms of people, VPDs and its prevention through vaccination?

Slums are different in many aspects. Slum inhabitants comprise of a floating population from different states and cultures and they live in overcrowded and unhygienic conditions. These are some of the crucial factors that affect VPDs and immunization. Necessities like employment, food, shelter, and clothing take priority over immunization.

One respondent stated, *“It was high time to plan immunization services by involving people living in the slums. Before vaccinating, understand their views which will help in building their trust. It should always be a win-win situation for them and us.”*

Other responded, *“Slums population is increasing rapidly. Challenges related to slums are different, so one must think differently to increase immunization coverage. This is also important to prevent any future outbreaks of VPDs.”*

#### 3.3.3 How do slums populations view vaccination- their perception about VPDs, vaccines and vaccination?

Though the situation has improved in last several years, there is a still a long way to go. This could be because of the inherent limitation associated with their economic status and the nature of the dwelling they reside in. Even in slums there are people who want their kids to be vaccinated and enquire about the various vaccines. Mass Campaigns like pulse polio immunization and Mission Indradhanush have played an important role in improving awareness related to immunization even in the slums.

#### 3.3.4 What are the reasons for vaccinating or not vaccinating?

The priorities are different for people living in slums. They have limited knowledge about the VPDs and its prevention through immunization. They are also afraid of the adverse effects. Most importantly they are on daily wages and when they leave for work, there is no one to take the child to the immunization centre. They can get their child vaccinated on Sunday or even on holidays, however, immunization days are usually between Monday to Friday. Polio was an exception as most of the campaigns were held on Sunday or on holidays.

#### 3.3.5 What are the major challenges?

Demand side:

• People are from diverse cultures
• Limited awareness about VPDs and vaccination
• Priority is to earn livelihood
• Less community participation thus immunization is still not owned by the people living in these settings.

Supply side:

• Top-down approach
• The responsibility is restricted only to vaccinate those who come for a visit
• No active follow-up and limited accountability
• Lack of on the job trainings and supportive supervision
• Limited resources including trained and dedicated manpower, poor infrastructure for storage of vaccines and other logistics (e.g. inactivated polio).

#### 3.3.6 What are the opportunities available to improve the coverage?

Demand side:

• Create awareness similar to pulse polio program
• Involve communities in planning and implementation of immunization
• Announcement of immunization drives from places of worship, especially, during festivals
• Use of public notice boards to inform about immunization
• Involve teachers and quacks
• Recall message (e.g. WhatsApp, mobile text message) one day prior to scheduled date
• Recognition or reward to those villages or communities having active participation and considerable immunization coverage.

Supply side:

• Know your area and population
• List beneficiaries
• Update immunization micro-plan each year
• List drop outs
• Outreach session for unreached or never reached
• Organize catch up rounds once in 3 months for dropout kids
• During pregnancy – start counselling regarding immunization benefits
• Ensure timely supply of vaccines
• On job training and supportive supervision
• Build positive attitude and behavior by motivating healthcare service providers.

Policy:

• Listen to your community and service providers
• Provide optimal resources
• Improve disease surveillance
• Conduct awareness program for staffs at regular intervals
• Hold immunization session at hours that are convenient for families
• Address patients' concerns
• Regular catch-up programs especially, on Sunday, public holiday or in evening
• Incentive to both beneficiaries and the service providers.

### 3.4 What are the expectations from beneficiary, community, service provider/policy maker?

A healthy life for every kid is possible only with the involvement of people at various levels. For example:

• Beneficiaries: Should participate in immunization program as it saves lives
• Community: Support the system to generate awareness and to vaccinate kids
• Service provider: Be accountable and provide services with personal attention
• Policy maker: Listen to field staffs and provide more supports e.g. resources
• Active involvement of political parties and local civic bodies to make immunization as a shared service
• Bring government, non-governmental organization (NGO), academic bodies, research institutions, professional bodies (Indian Academy of Pediatrics (IAP), Indian Medical Association (IMA) etc.), medical colleges, and civic bodies on a common platform to improve the immunization coverage in the sums

## 4 Discussion

Immunization has substantially contributed to reductions in global childhood morbidity and mortality due to VPDs (National vaccine policy, 2011; Sharma, 2007). India has a society which is multifaceted and differs virtually in every aspect of social life. A large part of this society resides in slum were the coverage is quite low (Singh, 2018). Governing immunization services to achieve desired outcome is complex as it involves interactions at multiple levels and in different contexts (GarcõÂa et al., 2014). Effective communication, interactions at different levels, behavior, logistic as well as financial support are some of the potential factors essential to strengthen routine immunization uptake (Smith et al., 2017). This study explored some of these factors that influence the routine immunization through observations and interactions with healthcare givers, influencers and policy makers.

Some crucial thoughts captured through the questions proposed in non-participatory observations revealed certain areas in which the health workers were doing good and others where they needed to focus more. There was no concern with respect to vaccine storage or its usage. The concern was around the communication and behavior of staff towards the beneficiaries, for e.g. providing inadequate information related to vaccine or making them to wait for long hours. Also, information displayed on the walls at immunization center was quite old. This can lead to serious implications in terms of misconception or not understanding the importance of vaccines, etc. Poor counselling and limited information with respect to benefits or adverse effects of immunization, scheduling, number of sessions, age at which immunization is started are some of the reasons that contribute to the problem of incomplete immunization (Tagbo et al., 2014; Rahman et al., 2012; Maina et al., 2013; Zewdie et al., 2016). In several qualitative studies it has been shown that mothers who are afraid of vaccine adverse effects, either decline or delay subsequent immunizations (Smith et al., 2017). Although, vaccines are well tolerated, no vaccine is entirely without risk. Thus, due to lack of awareness it has been seen that when few children experience mild adverse effects, their mothers may refuse further immunizations (Bofarraj, 2011; Abdulraheem et al., 2011; Juliet et al., 2011). Therefore, creating awareness and giving balanced information to the mothers forms an essential component of immunization.

Further, focus on behavior and attitude modification of healthcare workers is equally important. As seen in this study, influencers perceived health workers as unfriendly as well as unsupportive and therefore avoided them rather than consult or seek support even in situations where they didn't know what to do. A similar behavior or relationship have been previously reported in several developing countries. These problems were also compounded by long waiting time and poor service arrangements (Abdulraheem et al., 2011; Negussie et al., 2016). These may be attributed to the poor training or limited number of personnel in the facilities and consequent high workload. Thus, there is a need to improve the overall clinic environment and conduct regular training sessions for healthcare workers not only from a technical aspect but also in terms of enhancing their ability to communicate and create confidence in the beneficiaries.

Immunization is a shared responsibility involving community, healthcare service providers, policy makers, and parents who are active participants in the process. Effective communication at different levels and consideration of factors especially at the receiver end is essential to strengthen routine immunization uptake.

## Conflict of interest

SS and AA work for GlaxoSmithKline Pharmaceuticals, India. DS works for ICMR-National Institute of Medical Statistics, New Delhi, India. MDV works for National Polio Surveillance Project, India. This independent work is neither supported nor influenced by any associated institutions and is a part of PhD thesis project. The authors have no financial involvement with GlaxoSmithKline or any organizations with the subject matter or materials discussed in the manuscript apart from those disclosed.
